# An Engineered Nisin Analogue with a Hydrophobic Moiety Attached at Position 17 Selectively Inhibits *Enterococcus faecium* Strains

**DOI:** 10.1021/acschembio.4c00337

**Published:** 2024-09-10

**Authors:** Longcheng Guo, Oscar P. Kuipers, Jaap Broos

**Affiliations:** Department of Molecular Genetics, Groningen Biomolecular Sciences and Biotechnology Institute, University of Groningen, Groningen, 9747 AG, The Netherlands

## Abstract

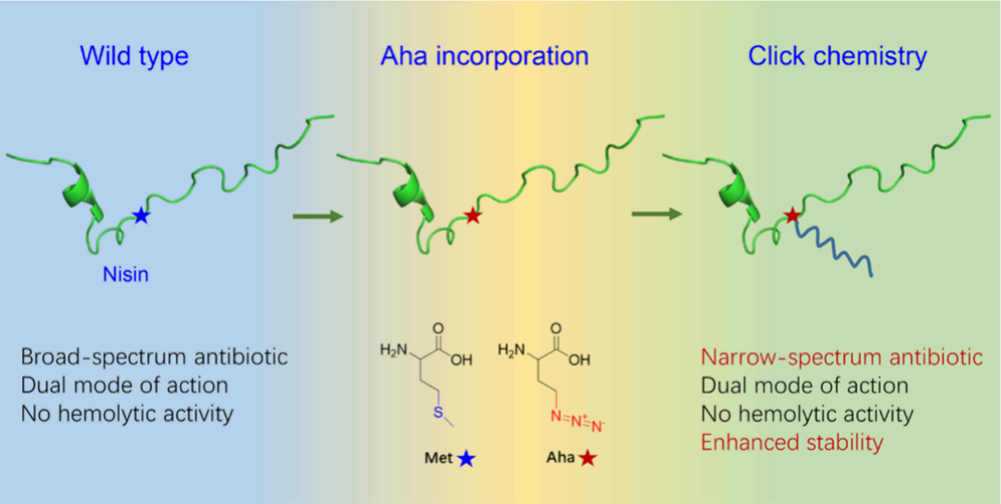

Antibiotic resistance is one of the most challenging global public health concerns. It results from the misuse and overuse of broad-spectrum antibiotics, which enhance the dissemination of resistance across diverse bacterial species. Antibiotics like nisin and teixobactin do not target an essential protein and employ a dual mode of action antibacterial mechanism, thereby being less prone to induce resistance. There is a need for the development of a potent narrow-spectrum dual-mode-acting antibiotic against human pathogens. Using nisin, a lantibiotic with potent antimicrobial activity against many pathogens, as a template, the unnatural amino acid azidohomoalanine was introduced at selected positions and subsequently modified using click chemistry with 14 alkyne-moiety containing tails. A novel nisin variant, compound **47**, featuring a benzyl group-containing tail, exhibited potent activity against various (drug-resistant) *E. faecium* strains with an MIC value (3.8 mg/L) similar to nisin, whereas its activity toward other pathogens like *Staphylococcus aureus* and *Bacillus cereus* was significantly reduced. Like nisin, the mode of action of compound **47** results from the inhibition of cell wall synthesis by binding to lipid II and nisin–lipid II hybrid-pore formation in the outer membrane. The resistance of compound **47** against proteolytic degradation is markedly enhanced compared to nisin. Like nisin, compound **47** was hardly hemolytic even at a very high dose. Collectively, a modified nisin variant is presented with significantly enhanced target organism specificity and stability.

## 1 Introduction

Antimicrobial resistance poses a significant threat to the public health. A report from the United Kingdom government predicts that, without the development of new antimicrobial strategies, antibiotic-resistant infections could lead to 10 million deaths worldwide annually by 2050.^1^ The acceleration of the rate of resistance development is attributed to the overuse and misuse of broad-spectrum antibiotics.^2^ These antibiotics not only naturally foster resistance but also promote the selection of resistance mechanisms in nontarget species, enabling their subsequent transfer to pathogenic bacteria. Additionally, the use of broad-spectrum antibiotics can disrupt the microbiota, which plays crucial roles in various aspects of human biology.^3^ To mitigate the risk of unintentional development of antibiotic resistance and microbiota disruption, employing a species-selective antimicrobial agent that specifically targets and eliminates the disease-causing strain is desired.

The issue of bacterial resistance is not uniformly distributed among all bacterial species.^4^ The Infectious Disease Society of America (IDSA) has identified six species with pronounced threats due to their potential mechanisms of multidrug resistance (MDR) and pathogenicity, collectively referred to as ESKAPE pathogens (*Enterococcus faecium*, *Staphylococcus aureus*, *Klebsiella pneumoniae*, *Acinetobacter baumannii*, *Pseudomonas aeruginosa*, and *E**nterobacter* species).^5^*E. faecium*, in particular, is globally linked to hospital outbreaks involving bacteremia, urinary tract infections, and endocarditis, among other conditions.^6^ These outbreaks not only impose significant economic burdens on healthcare systems but also pose a severe risk to susceptible patients, potentially leading to fatal infections.^7^ The challenge is further exacerbated by the complexities of treatment associated with the development of high-level resistance to various antibiotics, whether intrinsic or acquired through the horizontal transfer of plasmids and transposons.^6,8^ Vancomycin-resistant *E. faecium* (VRE) has emerged as the predominant multidrug-resistant *Enterococcus* species in healthcare settings.^9^ Consequently, there is an urgent need to discover new therapeutic agents and develop alternative approaches to address this highly challenging drug-resistant infection.

Protein-targeting antibiotics like penicillins are known to be most sensitive for inducing drug resistance.^10^ Antimicrobial peptides (AMPs) have emerged as a promising avenue for addressing antimicrobial resistance^11^ as many, for example, vancomycin and mersacidin, do not target a protein but lipid II, an essential building block for cell wall synthesis. Other AMPs, like teixobactin, target both lipid II and lipid III,^10^ whereas nisin targets lipid II, leading to the inhibition of the cell wall synthesis and forming nisin-hybrid pores in the cell membrane.^12^ Both teixobactin and nisin are very potent antimicrobials, and their dual mode of action on essential nonprotein targets renders the development of bacterial resistance challenging.^13,14^ Although many AMPs have proven efficacy, in many cases, they lack specificity and target a wide spectrum of Gram-positive and/or Gram-negative bacteria.^15,16^ To address this limitation, several research groups have employed a specifically targeted antimicrobial peptide strategy.^17−20^ The strategy involves constructing a hybrid peptide that combines two functionally independent components, namely, a targeting peptide and a broad-spectrum AMP, linked by a short flexible linker. The targeting peptide imparts selectivity to the AMP domain by binding to specific determinants on the pathogen’s surface, such as membrane charge, receptors, cell wall components, or distinctive virulent attributes.

Despite achieving some promising results, the method encountered certain limitations. Because of its gene-encoded character, nonpeptide molecular entities such as fatty acid tails or sugar groups cannot readily be included. Incorporating the desired moiety at positions other than those at the N- or C-terminus is not straightforward, while the termini of AMPs often play a crucial role in their function, making them unsuitable for modifications. Another limitation of the strategy is that these hybrid peptides are typically not well structured, making them susceptible for proteolytic degradation.^21^

In antibacterial nonribosomally produced lipopeptides,^22^ nature is using a hydrophobic tail to enhance the activity. As cell membrane architecture and lipid composition differ between pathogen species,^23^ engineering of the tail structure and its positioning within an AMP might be a strategy to enhance both activity and selectivity. Potent antibacterial nonribosomally produced lipopeptides like daptomycin contain ring structures and are rich in noncanonical amino acids, which make them more resilient to proteolytic degradation.^24^ Many antibacterial ribosomally synthesized and post-translationally synthesized peptides (RiPPs) are also stabilized by ring structures and amino acid modifications and have the advantage of engineering their structure using molecular biology techniques.^25^ One of the best studied examples is nisin, a 34-residue long RiPP containing five lanthionine rings.^14^ Koopmans et al. was the first to introduce a hydrophobic tail at the C-terminus of an antimicrobial RiPP.^26^ The low activity of a truncated nisin variant could be enhanced to wild-type nisin activity when a suitable tail was attached. In few other studies, similar results were observed using different RiPPs.^27,28^ In all of these studies, the tail was introduced at the N- or C-terminus.

**Figure 1:**
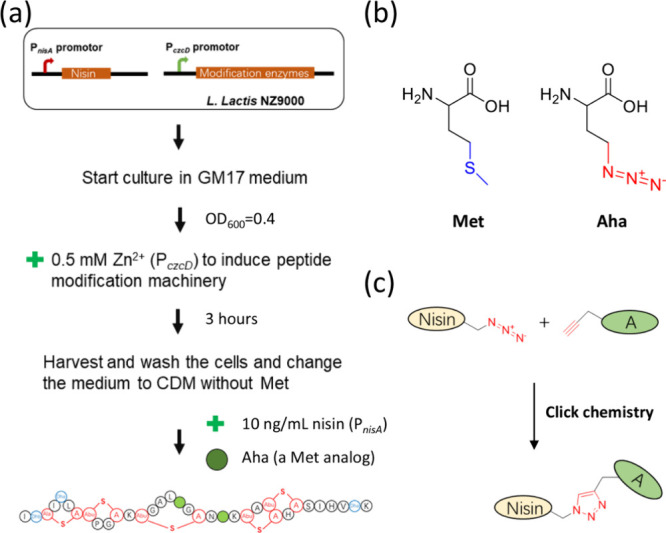
(a) Schematic overview of the force-feeding method for noncanonical amino acid Aha incorporation into nisin. Translation of the genes for the nisin modification machinery *nisBTC*, controlled by the P_*czcD*_ promoter, is initiated while cells are growing in rich medium. After the exchange of the growth medium to a chemically defined medium (CDM) without Met but supplemented with the Met analogue Aha, 10 ng/mL nisin (P_*nisA*_ promotor inducer) is added to start expression of single Met nisin construct. In this way, the analog Aha instead of Met is incorporated into nisin with high incorporation efficiency and yield, at the same time not affecting the NisBTC enzymes needed for the peptide postmodifications, e.g., dehydration and cyclization. (b) Structures of Met and its analog. Met, methionine; Aha, azidohomoalanine. The side chain difference is indicated in blue/red. Aha possesses a unique azide functional group capable of reacting with alkyne substrates. (c) Scheme of Aha-labeled nisin attached with alkyne substrates using the copper (Cu^+^)-catalyzed azide–alkyne click chemistry reaction.

Recently, we reported a *Lactococcus lactis*-based expression system for the efficient incorporation (>99.5%) of the Met analog azidoalanine (Aha) in nisin, together with a high expression yield of 9.5 mg/L pure peptide (Figure 1a).^29^ Aha possesses a unique azide functional group (Figure 1b) capable of reacting with alkyne substrates, commonly referred to as “click chemistry” (Figure 1c).^30^ This makes the system very suitable to introduce an alkyne moiety containing a tail molecule at a selected residue position in nisin, and this was demonstrated for a single alkyne (1-undecyne) introduced at four positions. In this study, we present 42 new nisin analogues by testing a diverse set of tail compounds, including linear and branched alkyl chains, phenyl groups, and polyprolines, introduced at four different positions of nisin. For these newly synthesized peptides, we report the antimicrobial activity profiles for a dozen pathogens, modes of action, stabilities, and toxicity toward human red blood cells.

## 2 Results and Discussion

### 2.1 The Impact of the Tail Structure on the Antibacterial Activity of the Nisin Constructs

**Figure 2:**
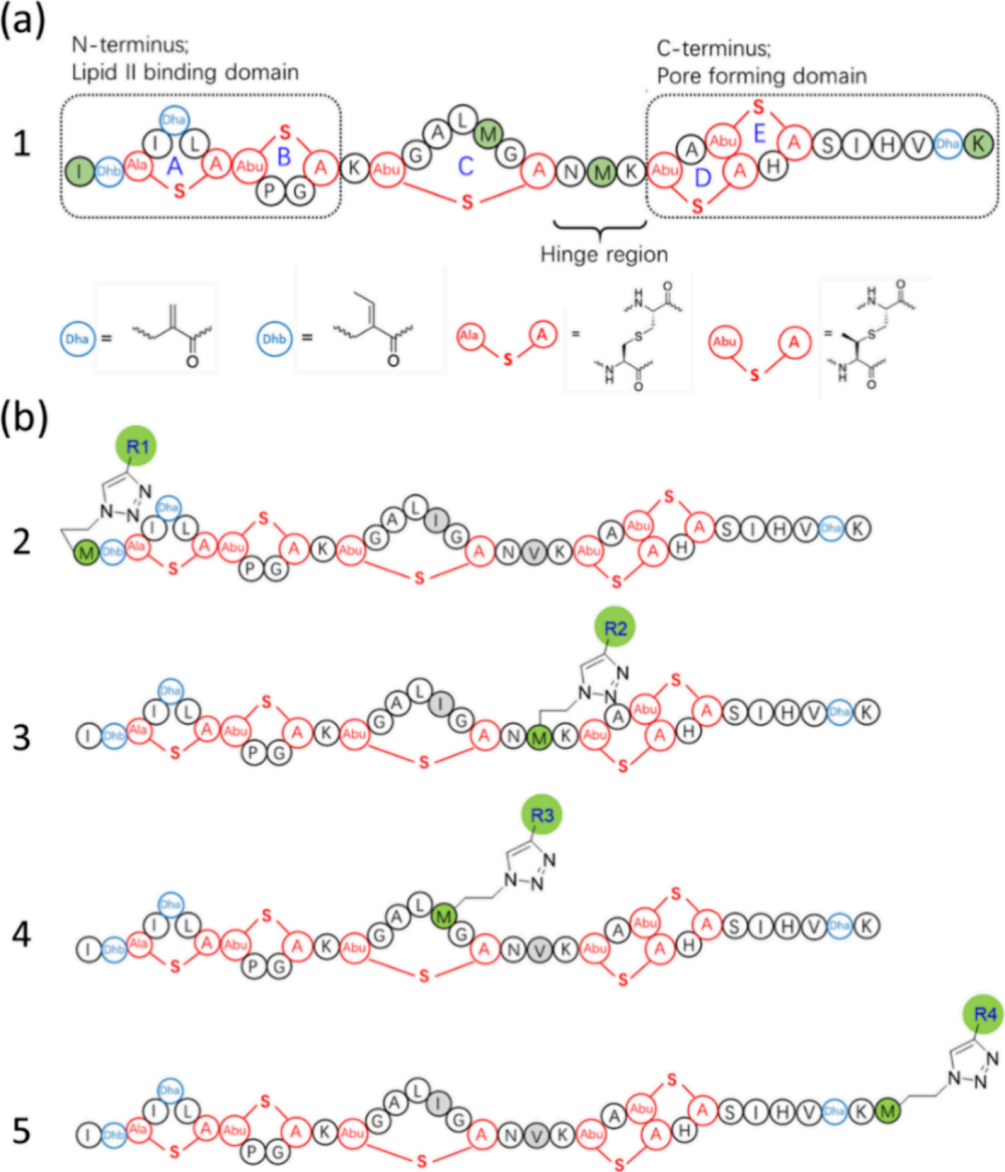
(a) Structure of nisin A. Dha, dehydroalanine; Dhb, dehydrobutyrine; Ala-S-A, lanthionine; Abu-S-A, methyllanthionine. The functional domains, including the lipid II binding site, pore formation domain, and hinge region, are indicated. The positions that will be chemically modified are indicated in green. (b) Structure of various nisin constructs used in this study and each labeled produced using click chemistry. In gray, Met replaced by Ile or Val; in green, position of Aha incorporation and subsequent modification by click chemistry.

Nisin (Figure 2a) is one of the best studied antimicrobial RiPPs and has been documented since 1928.^14^ Derived from various strains, nisin exhibits potent activity against Gram-positive bacteria including *Bacillus cereus*, *Listeria monocytogenes*, enterococci, staphylococci, and streptococci.^14^ Nisin is characterized by the presence of five (methyl)lanthionine rings and various post-translational modifications (Figure 2a). The dual functionality of nisin emanates from two functional parts located at the N- and C-termini, respectively.^31^ The N-terminal segment, hosting three post-translationally incorporated (methyl)lanthionine rings (A, B, and C), connects to the C-terminal rings (D and E) through a flexible hinge region comprising three amino acids (Figure 2a). The collective formation of the A, B, and C rings creates a “cage”, facilitating the binding of lipid II’s pyrophosphate moiety, thereby disrupting cell wall synthesis.^14^ This binding enhances the capability of the C-terminal segment, housing rings D and E, to generate pores in the cell membrane, resulting in the rapid efflux of ions and cytoplasmic solutes. Nisin’s distinctive dual mode of action contributes to its remarkable efficacy and plays a crucial role in limiting the emergence of nisin resistance.

**Figure 3:**
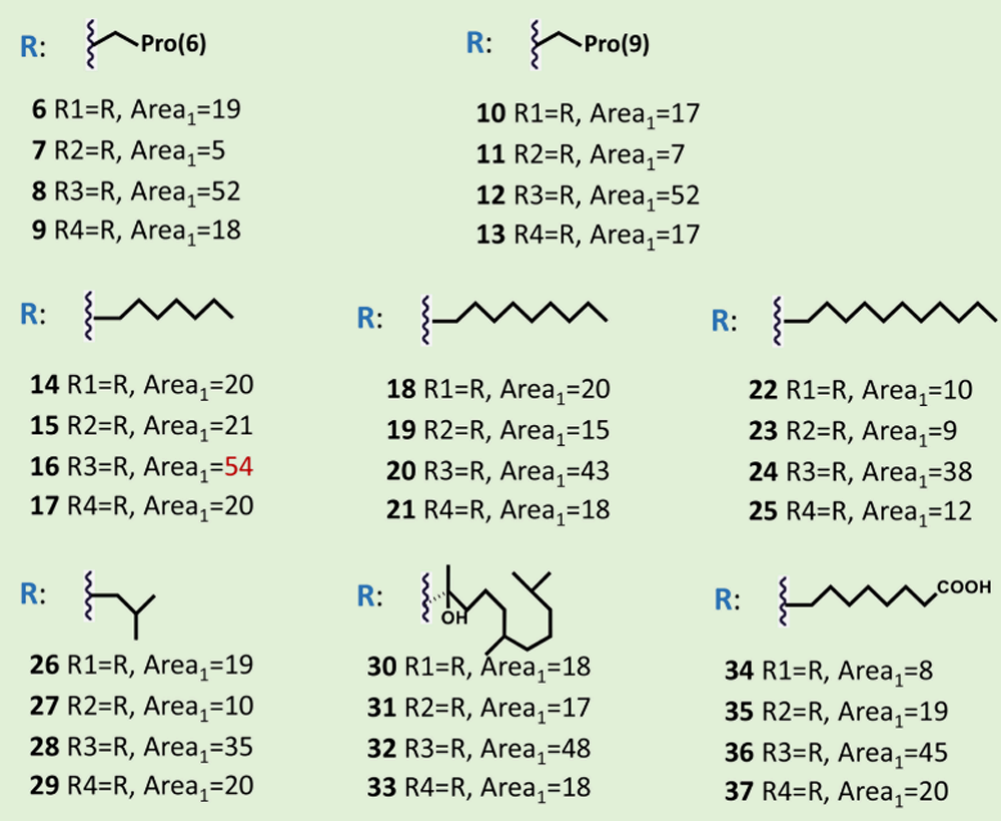
Antibacterial activities of nisin derivatives against *Enterococcus faecium* assessed through an agar well diffusion assay. Zone diameters were measured in millimeters, and the area of the inhibition zone (π*r*^2^) minus the well area (π*r*^2^) was calculated in mm^2^. The red color denotes the highest activity observed among the tested analogs. Experiments were performed in triplicate, and variations in zone diameters were 1 mm or less.

Recently, we presented a *L. lactis*-based expression system for the efficient labeling of four single-Met containing nisin mutants with Aha creating an attractive expression platform for subsequent labeling with an alkyne moiety containing tail molecule using click chemistry.^29^ Using this platform, in the first round of screening experiments, each single-Aha containing nisin construct was modified with eight different tail molecules, yielding compounds **6**–**37**, and their structures are presented in Figures 2 and 3. The screening assay using *E. faecium* distinctly shows that different side chains influence nisin’s antibacterial activity. The best modification site is Aha located at residue 17 (Figure 2b, no 4). When labeled with 1-decyne, this yielded compound **16,** for which the highest activity against *E. faecium* was observed. In compounds **18**–**21**, **22**–**25**, and **30**–**33**, for which 1-dodecyne, 1-pentadecyne, and 3,7,11-trimethyl-1-dodecyn-3-ol were used as tail molecule, a decreased antibacterial activity was observed (Figure 3). Lee et al., attaching different acyl chain lengths (C8, C10, C12, C14, and C16) to a nonapeptide, also observed that the longest tails yielded the lowest antibacterial activity.^32^ Comparing compounds **34**–**37** with compounds **14**–**17**, it is evident that introducing a carboxylic acid group at the end of the tail decreases the antimicrobial activity. Clicking with hydrophobic polyprolines yielded compounds **6**–**13**, which also showed good activity, just somewhat less potent than its counterparts containing more hydrophobic alkyl tails.

**Figure 4:**
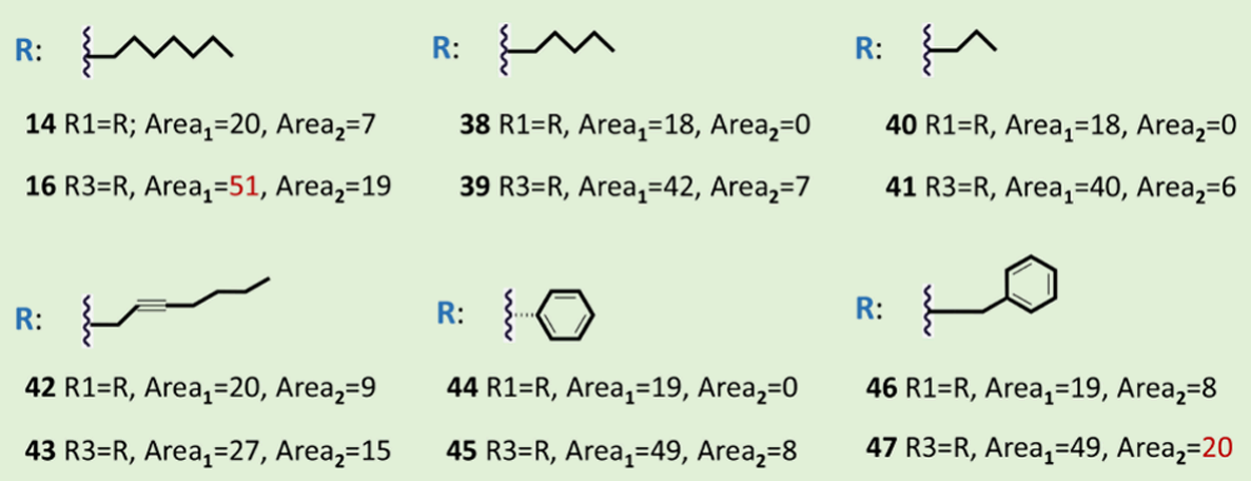
Antibacterial activities of nisin derivatives against *Enterococcus faecium* (labeled as Area_1_ [mm^2^]) and *Listeria monocytogenes* (Area_2_ [mm^2^]) evaluated using an agar well diffusion assay. The red color indicates the highest activity observed among the tested analogs against either *Enterococcus faecium* or *Listeria monocytogenes*. Experiments were performed in triplicate, and variations in zone diameters were 1 mm or less.

Because for all eight tested tail structures explored in the first round, the nisin variant with Aha at position 17 gave the best result, two shorter alkynes (1-hexyne and 1-octyne) and a monosaturated undecyne were chosen to be introduced only at this Aha position. Nisin with Aha at the first residue position was also chosen for further study. The click chemistry products were tested against two strains, *E. faecium* and *L. monocytogenes* (Figure 4). A lower activity against *E. faecium* was observed in this round for all variants created compared to the most active variant, compound **16**, discovered in the first round. Thus, shortening the chain length of compound **16** by two or four carbons lowers its activity (Figure 4). In this context, it is interesting to consider various clinically used lipopeptide antibiotics, including telavancin, dalbavancin, and daptomycin, that also contain chains of similar size as in compound **16**.^22^ Two tail compounds containing a phenyl ring were also tested. Best results were obtained with 5-phenyl-1-pentyne to create compound **47**, which shows a similar activity against *L. monocytogenes* as nisin and an activity comparable with compound **16** against *E. faecium* (Figure 4). Next, a more detailed investigation was conducted on compounds **14**, **16**, **46**, and **47**, which were formed by the click chemistry reaction between 1-decyne or 5-phenyl-1-pentyne and Aha at residue position 17 or at the N-terminus of nisin.

### 2.2 Selective Activity of Compounds **16** and **47** against *E. faecium* at Low Concentrations

**Figure 5:**
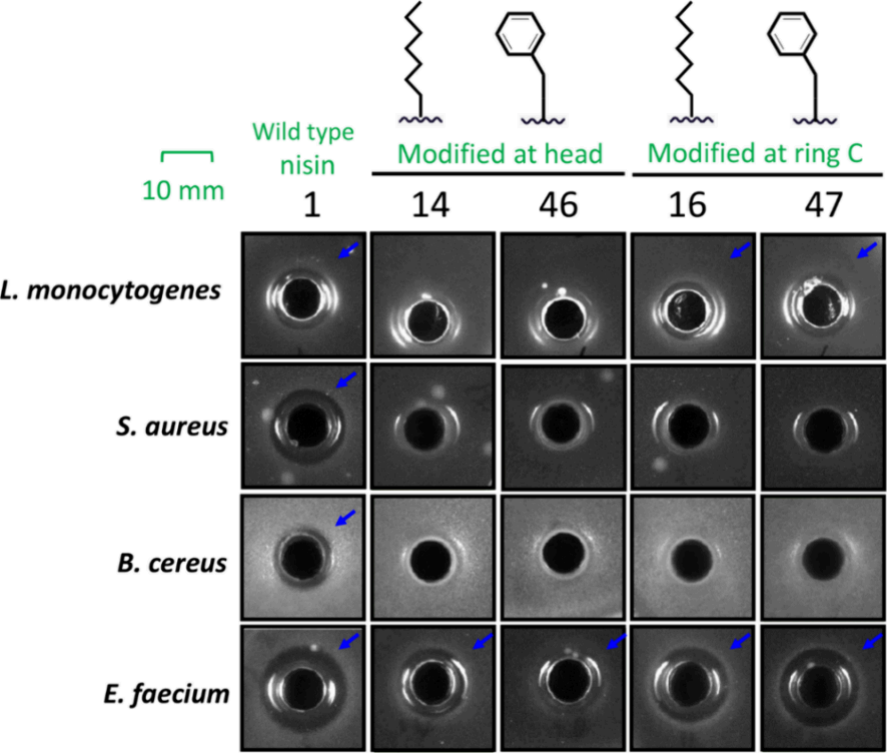
HPLC-purified nisin and nisin analogs tested against four pathogenic Gram-positive bacteria. Whereas wild-type nisin exhibits broad activity against all tested strains, the newly synthesized nisin variants demonstrate selective efficacy. The blue arrow indicates that the peptides exhibit antibacterial activity.

The four compounds mentioned above were purified (Figures S1 and S2) (see the Experimental Section) and tested, together with nisin for comparison, in agar well diffusion assays using four pathogenic Gram-positive strains, namely, *Staphylococcus aureus*, *Enterococcus faecium*, *Bacillus cereus*, and *Listeria monocytogenes*. As expected, wild-type nisin demonstrated broad-spectrum antibacterial activity (Figure 5). However, introduction of the tails at the first residue position of nisin, yielding compounds **14** and **46**, resulted in variants losing activity against *Staphylococcus aureus*, *Bacillus cereus*, and *Listeria monocytogenes* while retaining activity against *Enterococcus faecium*, albeit with weaker potency compared to wild-type nisin (Figure 5). Interestingly, a similar narrowing of activity spectrum was observed for compounds **16** and **47**, but the high potency of nisin against *Enterococcus faecium* was retained.

Next, the specificity of compounds **16** and **47** was investigated in more detail using a panel of strains, including five *Enterococcus faecium* strains, one *Enterococcus faecalis* strain, two *Listeria monocytogenes* strains, two *Staphylococcus aureus* strains, one *Bacillus cereus* strain, and a Gram-negative *Escherichia coli* strain. Against different *Enterococcus faecium* strains, nisin and compounds **16** and **47** demonstrated high potency, whereas they showed lower activity against the remaining tested strains compared to wild-type nisin (Table 1, Figure S3). In the MIC assay (Table 2), both compounds **16** and **47** displayed potent activity against *E. faecium*, with a low concentration of 3.8 μg/mL, the same as that for nisin. However, compounds **16** and **47** exhibited 4 times higher MIC values against *S. aureus* and *B. cereus* compared to nisin, whereas all three compounds show the same MIC value against *L. monocytogenes*, which required a higher concentration of 15.3 μg/mL. Collectively, the newly developed compounds **16** and **47**, modified at residue position 17 of nisin, exhibit a highly specific and potent lantibiotic activity against *E. faecium* strains.

**Table 1:**
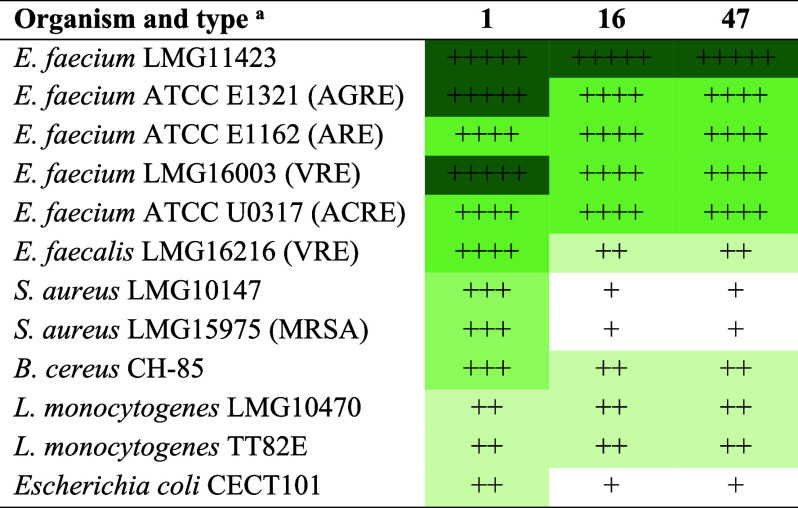
Antimicrobial Activity of Wild-Type Nisin (Compound **1**) and Compounds **16** and **47** against Pathogenic Microorganisms^b^

a AGRE, ampicillin-gentamicin-resistant enterococci; ARE, ampicillin-resistant enterococci; VRE, vancomycin-resistant enterococci; ACRE, ampicillin-ciprofloxacin-resistant enterococci; MRSA, methicillin-resistant *Staphylococcus aureus*.

b Note: The diameter (mm) was determined using the spot-on-lawn assay (Figure S3) in triplicate, with corresponding ratings as follow: 0–4 mm, + ; 4–8 mm, + +; 8–12 mm, +++; 12–16 mm, ++++; and 16–20 mm, +++++. A darker color indicates a higher level of antibacterial activity.

**Table 2:** Antimicrobial Profile of Nisin (**1**) and Compounds **16** and **47** against Selected Gram-Positive Strains^a^

organism and type	MIC (μg/mL)		
	**1**	**16**	**47**
*Listeria monocytogenes* LMG10470	15.3	15.3	15.3
*Bacillus cereus* CH-85	7.7	30.6	30.6
*Staphylococcus aureus* LMG15975 (MRSA)	3.8	>15.3	15.3
*Enterococcus faecium* LMG16003 (VRE)	3.8	3.8	3.8

a VRE, vancomycin-resistant enterococci; MRSA, methicillin-resistant *Staphylococcus aureus*. Presented MIC values are based on three experiments, yielding the same outcome for all tested conditions.

### 2.3 Compound **47** Demonstrates Low Hemolytic Activity

**Figure 6:**
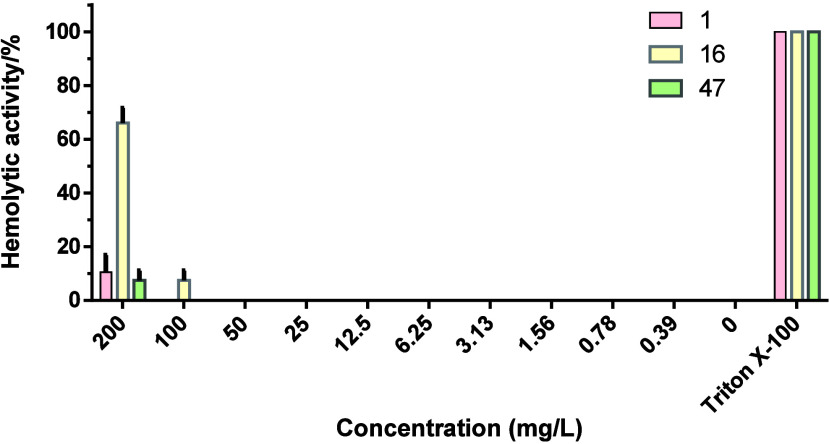
Hemolytic activity of nisin and two newly synthesized nisin variants. Human erythrocytes were incubated with nisin (**1**), compound **16**, and compound **47** at concentrations ranging from 0.39 to 200 mg/L. Hemolytic activity was evaluated based on hemoglobin release. Cells treated without any tested compound served as the no lysis control, whereas cells treated with 10% Triton X-100 were used as completely lysed. The data represent three independent experiments and the standard deviation is indicated.

Considering the potential therapeutic application of a bacteriocin, ensuring its safety is of critical importance. Previous studies have identified hemolytic activities in certain bacteriocins, such as listeriolysin S from *Listeria monocytogenes*(33) and cytolysin from *Enterococcus faecalis*.^34^ Koopmans et al. reported that attaching fatty acid tails to the C-terminus of a nisin fragment, nisin(1–12), resulted in semisynthetic lipopeptides displaying antibacterial activities on par with that of the wild-type nisin, whereas an increase in hemolytic activities was observed.^26^ Therefore, a close evaluation of the safety of new bacteriocins is essential. In this study, a hemolytic activity assay was conducted to assess the safety of compounds **16** and **47**. In this assay, human blood cells were incubated with varying concentrations of compound **16**, compound **47**, and nisin, ranging from 0.39 to 200 mg/L. Following a 1 h incubation at 37 °C, we measured the OD_414_ of the supernatants, and hemolytic activities were calculated, with 10% Triton X-100 serving as a positive control. Notably, both compound **47** and nisin exhibited minimal hemolytic activities (below 5%) at the high concentration of 200 mg/L. In contrast, compound **16** demonstrated 65% hemolytic activity at 200 mg/L and 5% hemolytic activity at 100 mg/L. However, all three peptides showed no hemolytic activity against human red blood cells at concentrations below 100 mg/L (Figure 6). It is interesting to note that the linear tail caused higher hemolytic activities (compound **16**) compared to the phenyl-moiety containing tail (compound **47**); at the same time, it did not influence the overall activity. In summary, the selective and potent antimicrobial compound **47** against *Enterococcus faecium* shows minimal hemolytic activity at 200 mg/L, a concentration 50-fold higher than its MIC value against this pathogen.

### 2.4 Compound **47** Exhibits Superior Proteolytic Resistance Compared to Nisin

**Figure 7:**
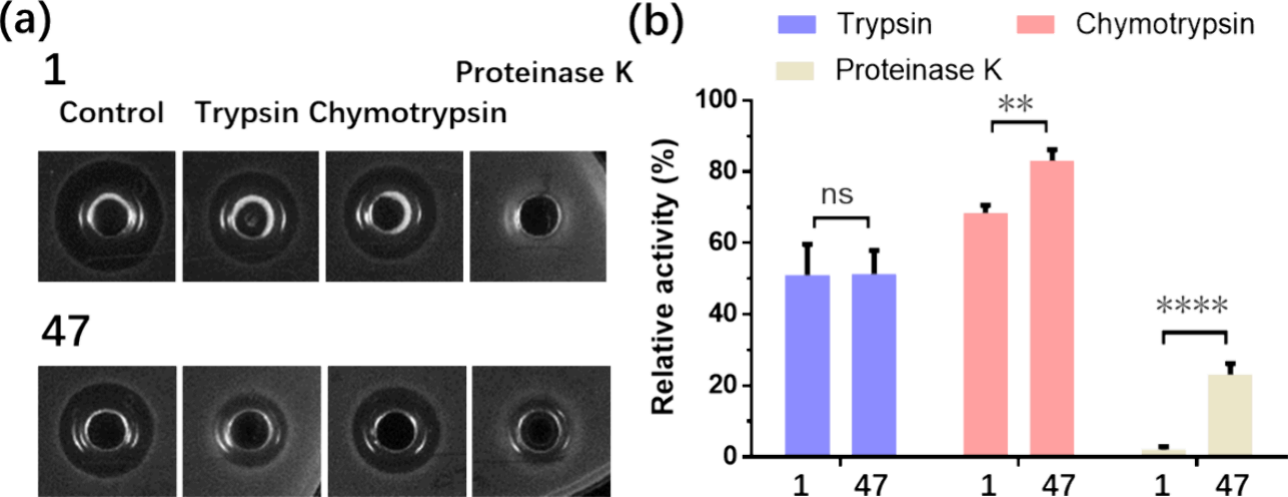
Investigation of the proteolytic stability profiles of nisin (compound **1**) and compound **47**. (a) Antimicrobial activity of the peptides evaluated against trypsin, chymotrypsin, and proteinase K. A representative image from three independent experiments is shown, with a control where no enzymes was added. (b) Relative antimicrobial activity of the peptides after exposure to different proteolytic enzymes. The relative activity was calculated as the area (enzyme added) divided by the area (control) multiplied by 100%. The standard deviation is indicated.

RiPPs have gained widespread applications, partly due to their resilience in harsh environments.^35^ To evaluate the stability of compound **47**, we exposed the peptide to different temperature values (Figure S4) and proteolytic enzymes (Figure 7).

The stability of compound **47** and nisin was assessed across temperatures ranging from 20 to 90 °C (Figure S4). Less than 40% loss in activity was observed for the higher temperatures over an 8 h storage period (Figure S4). This suggests a good stability at physiological temperatures and being suitable for applications like high-temperature pasteurization.^36^

Next the resistance against the proteolytic enzymes trypsin, chymotrypsin, and protease K was investigated by adding these enzymes in separate experiments to assay wells followed by overnight incubation at 30 °C. Nisin exhibited a different resistance against trypsin and chymotrypsin degradation, with 32 and 49% activity loss, respectively (Figure 7a,b). Compound **47** showed a similar profile as nisin when exposed to trypsin degradation but demonstrated improved resistance to chymotrypsin compared to nisin, with only a 16% loss in activity. Particularly when exposed to proteinase K, compound **47** exhibited an 77% loss in activity, whereas proteinase K completely eradicated nisin activity (Figure 7b).

The peptidic character of nisin imposes certain constraints, notably its vulnerability to proteolytic degradation in vivo,^37^ thereby restricting its broad applications. In previous studies, genome mining methods were utilized to discover new short nisin variants^29,38^ while employing a dehydrated amino acid engineering approach and a hybrid peptide, both aimed at enhancing its stability against proteases.^39,40^ In this study, attaching a hydrophobic tail was introduced as another strategy to enhance resistance to proteolytic enzymes. Previous studies have shown that lipidation can increase the stability of AMPs by blocking vulnerable areas to proteases or forming supramolecular structures, thereby prolonging the drug effect time of lipidated AMPs.^41^ For example, Chionis et al. synthesized lipidated anoplin with fatty acid chains incorporated at the N-terminus, retaining antimicrobial activity even after a 4 h exposure to trypsin.^42^ Another study by Zhong et al. found that lipid-modified anoplin analogs exhibited high stability toward trypsin hydrolysis, and its antimicrobial activity was not significantly reduced after preincubation in mouse serum.^43^ These findings highlight lipidation as a viable strategy to enhance the resistance of AMPs to protease degradation. In conclusion, compound **47** demonstrated high thermal stability and robust resistance to proteolytic enzymes.

### 2.5 Compound **47** Exhibits a Dual Mechanism against Bacteria Similar to Nisin

**Figure 8:**
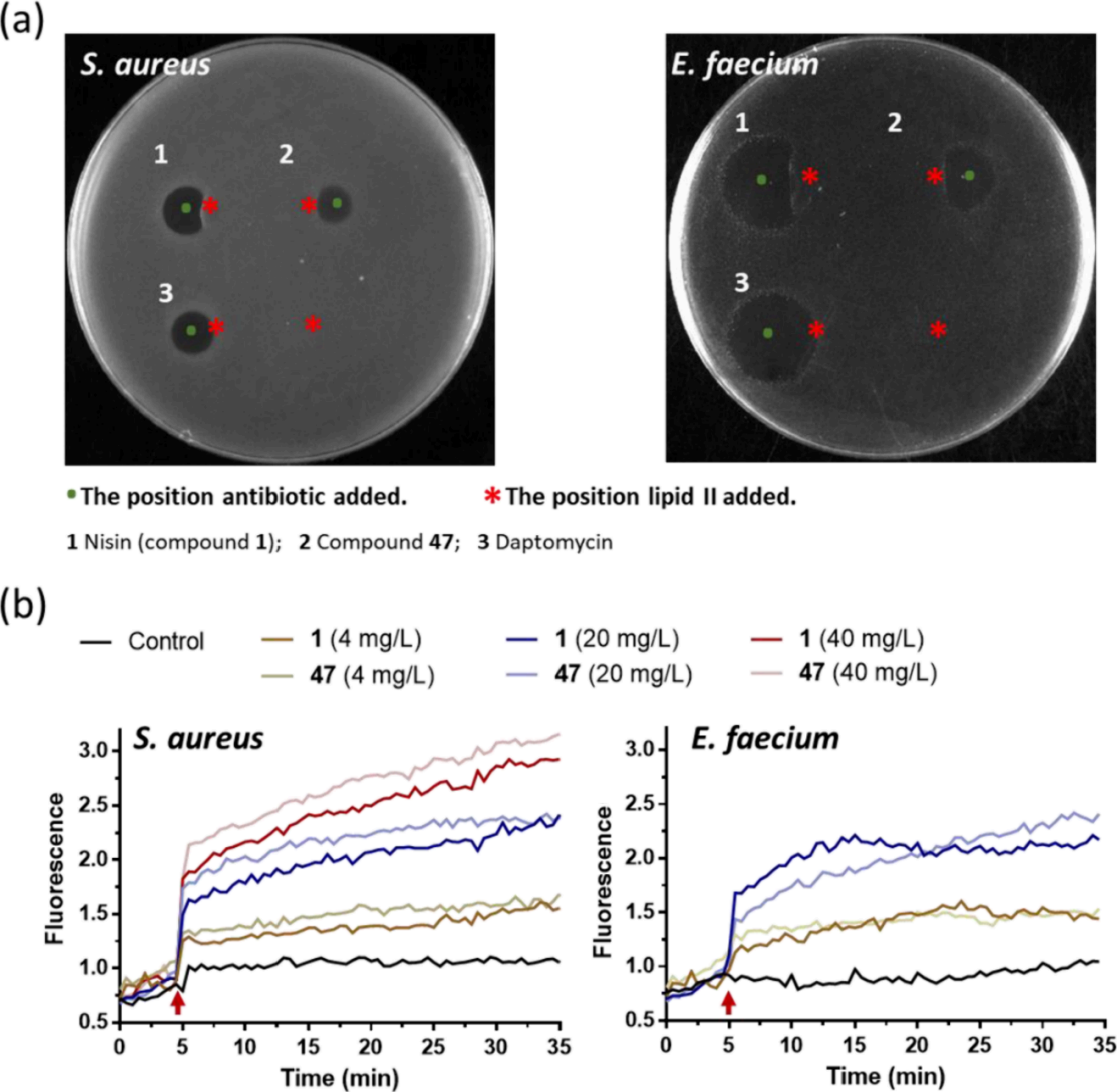
Mode of action of nisin (compound **1**) and the benzyl group conjugated nisin variant (compound **47**) against *E. faecium* and *S. aureus*. (a) A spot-on-lawn assay to assess the ability to bind to the cell wall synthesis precursor lipid II. Nisin was used as positive control, and daptomycin was used as negative control. 1, nisin; 2, compound **47**; and 3, daptomycin. *The position lipid II was added (300 μM, 2 μL). (b) Potassium leakage, as detected by the increase in fluorescence of PBFI probe, after addition of different concentrations antimicrobials. At 5 min, antibiotics were added.

Nisin exerts its antimicrobial effects through pore formation and inhibition of cell wall synthesis by specifically binding to lipid II, a crucial precursor in peptidoglycan biosynthesis.^14^ To investigate the impact of the attached benzyl group in compound **47** on the mode of action compared to nisin (compound **1**), we assessed its binding ability to lipid II (Figure 8a). Externally added purified lipid II reduced the antimicrobial activity of both nisin and the nisin variant against *E. faecium* and *S. aureus*, disrupting the typically circular antibiotic-induced halo. The non-lipid II-binding antibiotic daptomycin maintained its antimicrobial activity against the tested strains after the addition of purified lipid II, resulting in a circular halo. Despite structural modifications, compound **47** retained its ability to bind to lipid II, similar to that of nisin.

To explore the potential impact of modifications on nisin’s pore-forming activity, we conducted potassium ion efflux assays (Figure 8b). At the lowest concentration tested (4 μg/mL), both peptides exhibited a slight potassium leakage. Higher lantibiotic concentrations resulted in stronger leakage for both wild-type nisin and the nisin variant. Interestingly, at the same concentration, nisin and its variant demonstrated comparable efficiency for potassium leakage against the two strains (Figure 8b) despite differences in their antibacterial efficacy (Table 2). In conclusion, the benzyl group modification to nisin did not influence its binding ability to lipid II. Furthermore, its pore-forming ability against *E. faecium* and *S. aureus* was retained, with efficiency unaffected, despite exhibiting different profiles against the two tested strains. Our understanding of the reasons for the different antimicrobial activities of RiPPs in various microorganisms is still developing, and this also applies to intensively studied nisin. Because the mechanism of action of the narrow-spectrum compound **47** parallels that of nisin, it is conceivable that variation in cell wall thickness and/or composition between bacterial strains has a significant impact on the effectiveness of these antimicrobial agents.

## 3 Conclusions

Nisin, a broad-spectrum lantibiotic active against many Gram-positive bacteria, was synthetically modified to yield derivatives with a more focused antibacterial spectrum and enhanced proteolytic stability. The described approach offers a streamlined method for producing such compounds. Notably, among the analogues synthesized, compound **47** demonstrated the most potent antibacterial activity, particularly against drug-resistant strains such as VRE. Their distinct mode of action and superior stability render these semisynthetic lipopeptides promising candidates for further refinement and development as innovative specific antibiotics.

## 4 Experimental Section

### 4.1 Expression and Purification of Aha-Incorporated Nisin

*L. lactis* NZ9000 cells containing the *nisBTC* plasmid with the plasmid harboring the nisin gene were plated on GM17 agar plates supplemented with 5 μg/mL chloramphenicol (Cm) and erythromycin (Em) and cultured overnight at 30 °C. A selected colony was inoculated into 25 mL of GM17CmEm medium for initial growth followed by transfer to 1 L of the same medium. At an OD_600_ of approximately 0.4, 0.5 mM ZnSO_4_ was introduced to induce the expression of the modification machinery NisBTC. After 3 h, cells were triple-washed with phosphate-buffered saline (PBS) buffer (pH 7.2) and resuspended in 1 L of CDM-P medium lacking tryptone^29^ and Met. Aha (50 mg/L) and 10 ng/mL nisin were added for peptide expression. Following overnight growth, the supernatant was harvested through centrifugation at 8000*g* for 10 min.

The supernatant's pH was adjusted to 7.0, and it was incubated with purified NisP^29^ at 37 °C for 3 h to cut off the leader; then the supernatant was applied to a C_18_ open column (Spherical C_18_, 5 g, particle size: 40–75 μm, Sigma-Aldrich). The column was washed with 40 mL of different concentrations (25, 30, 35, 40, and 60%) of buffer B (buffer A, distilled water with 0.1% TFA; buffer B, acetonitrile with 0.1% TFA). The active fractions were lyophilized and further purified using an Agilent 1200 series high-performance liquid chromatograph (HPLC) equipped with a C_18_ column (NUCLEODUR C_18_ HTec, 5 μm, 250 × 4.6 mm, MACHEREY-NAGEL). The peak with correct molecular weight was collected, lyophilized, and stored at 4 °C until further use.

### 4.2 Click Reaction Protocol

Stock solutions of CuSO_4_ (100 mM), sodium ascorbate (1 M), and BTTAA (2-(4-((bis((1-*tert*-butyl-1*H*-1,2,3-triazol-4-yl)methyl)amino)methyl)-1*H*-1,2,3-triazol-1-yl)acetic acid, 50 mM) were prepared. A 200 μg sample of the Aha-labeled nisin variant was dissolved in a 0.05% acetic acid solution (pH 4.0, final reaction volume: 0.2 mL), and 1 equiv of alkyne compound was added to the solution. Subsequently, a premix of CuSO_4_ (4 μL) and BTTAA (40 μL) from stock solutions was added followed by the addition of 20 μL of sodium ascorbate. The reaction was performed at 37 °C for 1 h. The yields of four click chemistry reactions were determined using analytical high-performance liquid chromatography (HPLC) (Figure S1a), and the yields varied between 45 and 68%.

For the most potent analogs identified in this work, namely, compounds **14**, **16**, **46**, and **47**, the reaction volume was scaled up, and the compounds were purified using high-performance liquid chromatography (HPLC). Mass spectra of these purified compounds are presented in Figure S1b. To confirm that this procedure yields the modified nisin and at high purity, HPLC analysis of purified **47** was performed (Figure S2). Based on the area of the peaks, the purity of **47** is 96%. Subsequently, 30 μL of 0.1 mg mL^–1^ purified compound was added to the well to assess their activity.

### 4.3 Screening the Antibacterial Activity of Clicked Peptides by a Agar Well Diffusion Assay

Cultures grown overnight were introduced into 0.8% LB agar for *Listeria monocytogenes* or GM17 agar for *Enterococcus faecium* at a temperature of 45 °C, reaching a final concentration of 0.1% (v/v). Subsequently, 30 mL of the mixture was poured onto the plate. Upon solidification of the agar, 8 mm wells were created, and 30 μL of the aforementioned clicked solutions was spotted into the wells. The agar plate underwent overnight incubation at 37 °C followed by measurement of inhibition zones. By comparing the antimicrobial impact of the click chemistry products in this screening assay, we assume that the yields of the different reactions are similar. Zone diameters were recorded in millimeters, with the zone area (π*r*^2^) minus the well area (π*r*^2^) measured in millimeters.^44^

### 4.4 Minimal Inhibitory Concentration Assay

Minimal inhibitory concentration (MIC) values were determined through broth microdilution following standard guidelines.^29^ The inoculum was adjusted to approximately 5 × 10^5^ CFU/mL, and the MIC was characterized as the lowest concentration of the HPLC purified antimicrobial compound that exhibited no visible growth after overnight incubation at 37 °C.

### 4.5 Hemolysis Assay

Erythrocytes were sourced from a healthy human volunteer donor, and whole human blood was subjected to centrifugation at 600*g* for 15 min. Subsequently, plasma was removed, and the erythrocytes were subjected to three washes with PBS (pH 7) by centrifugation at 600*g* for 15 min each. After the supernatant was discarded, the packed cells were stored on ice. HPLC purified peptides were then introduced at final concentrations of 200, 100, 50, 20, 25, 12.5, 6.25, 3.13, 1.56, 0.76, and 0.39 mg/L in PBS containing 2% (v/v) erythrocytes. The cells were incubated at 37 °C for 1 h and subsequently centrifuged for 5 min at 800*g*. The supernatant was transferred to a 96-well plate, and absorbance was measured at a wavelength of 414 nm using a Thermo Scientific Varioskan LUX multimode microplate reader. The absorbance, relative to the positive control treated with 10% Triton X-100, was defined as the percentage of hemolysis.

### 4.6 Effects of Proteolytic Enzymes and Temperature on the Antibacterial Activity of Nisin and Compound **47**

The impact of proteolytic enzymes and temperature on antimicrobial activity was investigated using the representative strain *L. lactis* MG1363 through the agar well diffusion assay. Thirty microliters of the HPLC purified peptide (1 mg mL^–1^) was directly introduced into the agar well containing a final concentration of 1 mg mL^–1^ proteolytic enzymes (pH 7.2) or no proteolytic enzyme (control). The plates were incubated overnight at 30 °C followed by the measurement of inhibition zones. Temperature stability was assessed by incubating the peptide at 22, 55, 70, and 90 °C for the specified duration.

### 4.7 Spot-on-Lawn Assay to Measure Peptide–Lipid II Complex Formation

To assess the interaction between the HPLC purified peptide and lipid II, an overnight culture was introduced into 0.8% GM17 medium for *E. faecium* or LB medium for *S. aureus* (w/v, temperature 45 °C) at a final concentration of 1% (v/v). Subsequently, this mixture was evenly distributed onto 10 mL plates. The binding affinity of the peptide and lipid II was further examined by applying purified lipid II (300 μM, 2 μL) to the periphery of the antibiotic inhibition halo. Briefly, HPLC purified antimicrobials were initially applied to the agar plate, and once the antimicrobial solution drops had dried, lipid II was spotted along the edge of the inhibition halo. The plates were then incubated overnight at 37 °C.

### 4.8 Potassium Ion Efflux Assays

For the K^+^ release assay, the K^+^-specific fluorescent probe PBFI was employed. Strains of *E. faecium* were cultured in GM17 medium, whereas *S. aureus* strains were cultured in LB medium until reaching an OD_600_ of 0.6. Subsequently, cells were harvested (5000*g*, 5 min) and washed twice with 10 mM HEPES buffer (pH 7.2) containing 0.5% glucose. The washed cells were then resuspended in the same buffer supplemented with 10 μM PBFI. Using a Varioskan LUX Multimode Microplate Reader, data were acquired by exciting cells at 346 nm, and the fluorescence emission was measured at 505 nm to establish a baseline signal. Following this, varied concentrations of HPLC purified antibiotics were added, and the data were collected. Nisin served as a positive control in these experiments.

### 4.9 Statistical Analysis

Data analysis was performed using GraphPad Prism 6 (GraphPad Software, CA, USA). Differences in means (*P* ≤ 0.05) were determined using two-way analysis of variance (ANOVA) multiple comparisons whereby ns, *, **, ***, and **** corresponded to *P* > 0.05, *P* < 0.05, *P* < 0.01, *P* < 0.001, and *P* < 0.0001, respectively.

## Data Availability

All data supporting the findings of this study are available within the paper and its Supporting Information files.

## Supporting Information Available

Supplementary experimental section; mass spectra (MS) of purified nisin variants; HPLC profile of purified compound **47**; spot-on-lawn assay for the compounds **1**, **16**, and **47** antibacterial spectrum; and thermal stability profile of compounds **1** and **47** (PDF)
