# Supplementary material for: An Engineered Nisin Analogue with a Hydrophobic Moiety Attached at Position 17 Selectively Inhibits Enterococcus faecium Strains
Source: ACS Chem Biol. 2024 Sep 10;19(9):2023–31. doi: 10.1021/acschembio.4c00337 (PMC11420946; doi:10.1021/acschembio.4c00337)
Supplement: Supplementary file 1 — cb4c00337_si_001.pdf [file cb4c00337_si_001.pdf]

Supporting Information for

An engineered nisin analogue with a hydrophobic moiety attached at position 17 selectively inhibits *Enterococcus faecium* strains

Longcheng Guo[a], Oscar P. Kuipers[a], Jaap Broos[a],[*]

a Department of Molecular Genetics, Groningen Biomolecular Sciences and Biotechnology Institute, University of Groningen, Groningen 9747 AG, the Netherlands

***** Correspondence to Jaap Broos (j.broos@rug.nl).

Running title: Conjugated nisin variants

Supplementary Experimental Section

Materials

Reagents employed in molecular biology experiments were obtained from Thermo Fisher Scientific (Waltham, MA), and unless specified otherwise, all chemicals were acquired from Sigma-Aldrich (St. Louis, MO). L-azidohomoalanine (Aha), a methionine analog, was obtained from Iris Biotech GmbH (Marktredwitz, Germany). Whole human blood was ordered from Sanbio B.V., the Netherlands, and peptides were synthesized by Pepscan B.V., the Netherlands.

The alkyne moiety containing tails were bought from Sigma-Aldrich..

Name	CAS no.	Synthetic compound
1-Decyne	764-93-2	**14, 15, 16, 17**
1-Dodecyne	765-03-7	**18, 19, 20, 21**
1-Pentadecyne	765-13-9	**22, 23, 24, 25**
5-Methyl-1-hexyne	2203-80-7	**26, 27, 28, 29**
3,7,11- Trimethyl-1-dodecyn-3-ol	1604-35-9	**30, 31, 32, 33**
10-Undecynoic acid	2777-65-3	**34, 35, 36, 37**
1-Octyne	629-05-0	**38, 39**
1-Hexyne	693-02-7	**40, 41**
1,5-Decadiyne	53963-03-4	**42, 43**
Phenylacetylene	536-74-3	**44, 45**
5-Phenyl-1-Pentyne	1823-14-9	**46, 47**

Mass spectrometry

A 1 µL aliquot of the peptide was applied onto the target, allowed to dry, and subjected to multiple washes with Milli-Q water. Subsequently, an equivalent volume of matrix solution (5 mg/mL α-cyano-4-hydroxycinnamic acid dissolved in 50% acetonitrile containing 0.1% trifluoroacetic acid) was deposited onto the sample. Mass spectra (MS) were acquired using an Applied Biosystems 4800 Plus matrix-assisted

laser desorption/ionization time-of-flight analyzer (MALDI-TOF) operating in linear mode with external calibration.

Purification of the clicked products

Following the completion of click reactions, the reaction mixture was quenched with 0.8 mL of buffer (H_2O: acetonitrile, 5:95 +0.1% TFA), filtered through a 0.22 μm pore size membrane, and purified via HPLC using the following conditions: RP C_{18} column (4.6 × 250 mm), solvent A (H_2O with 0.1% TFA), solvent B (acetonitrile with 0.1% TFA), and a flow rate of 1 mL/min for a 42-minute run. A gradient protocol (0-7 min 28% solvent B, 7-30 min linear from 30% to 60% solvent B; 30-35 min 95% solvent B, and 35-42 min 28% solvent B) was employed. Fractions containing the target product were subsequently lyophilized.

Spot-on-lawn assay to test the peptides antibacterial spectrum

To evaluate the antibacterial spectrum of the peptides, an overnight culture of diverse strains was inoculated into the agar medium at 42°C, and the resulting mixture was dispensed onto plates at a volume of 30 mL per plate. Subsequently, 5 μl of the HPLC purified peptides (100 mg/L) was deposited onto the plates, and once the drops were dry, the plates were incubated overnight at 37°C.

(a)

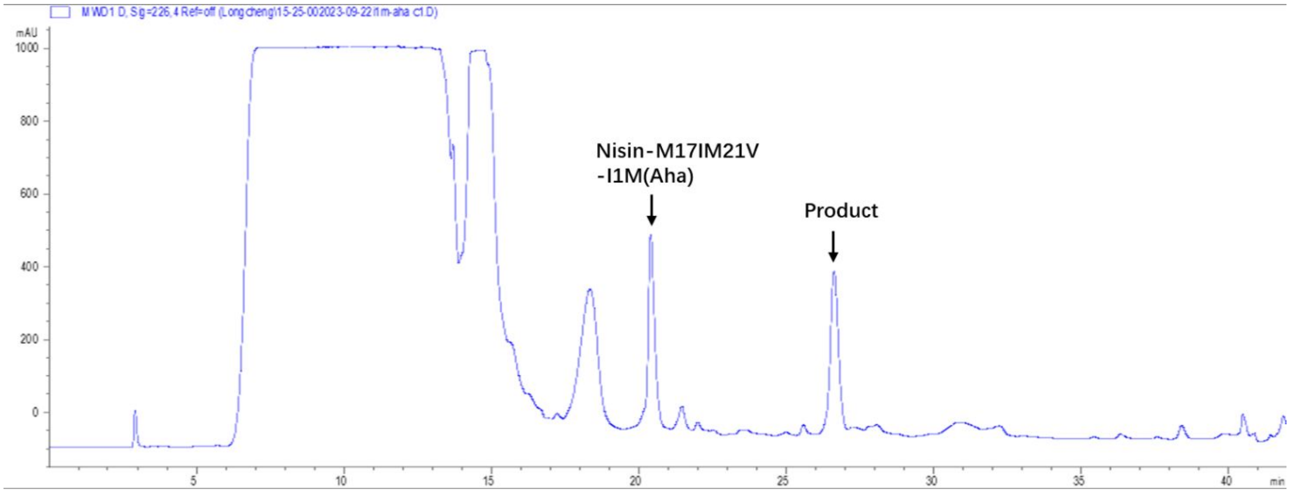

Compound **14**: Analytical RP-HPLC

(b)

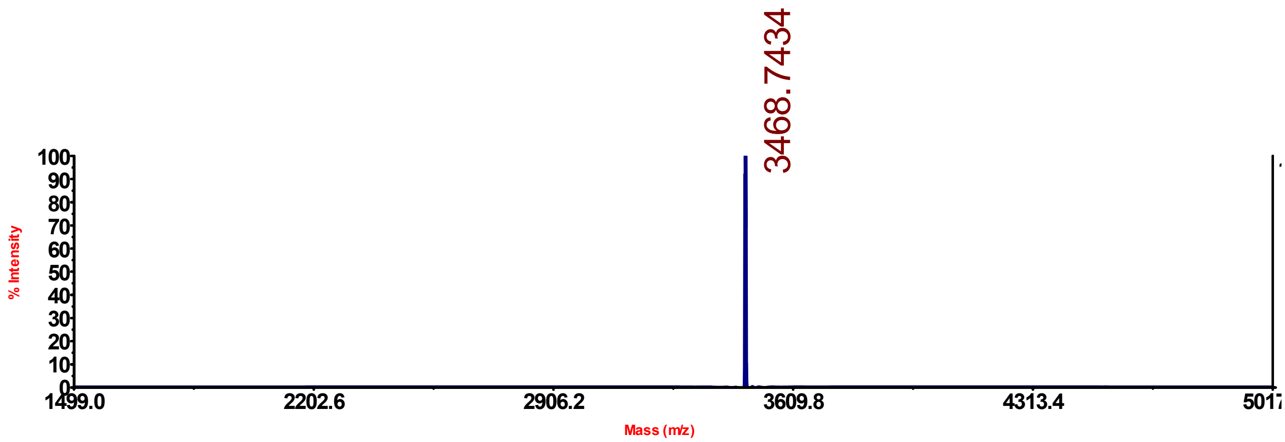

Compound **14**: MS

(a)

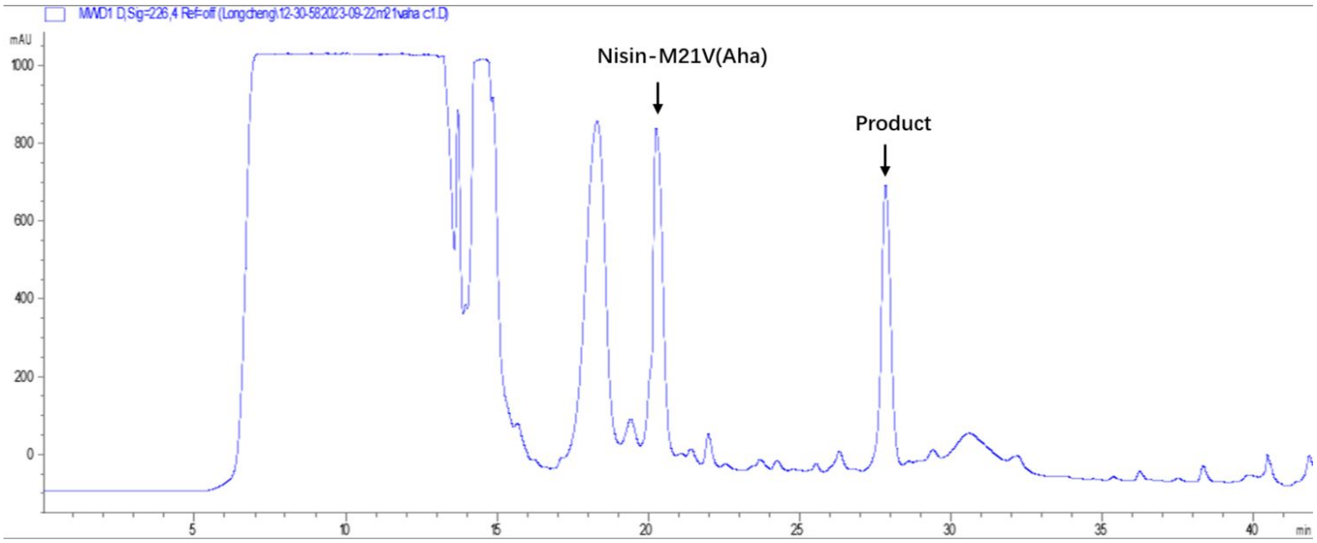

Compound **16**: Analytical HPLC

(b)

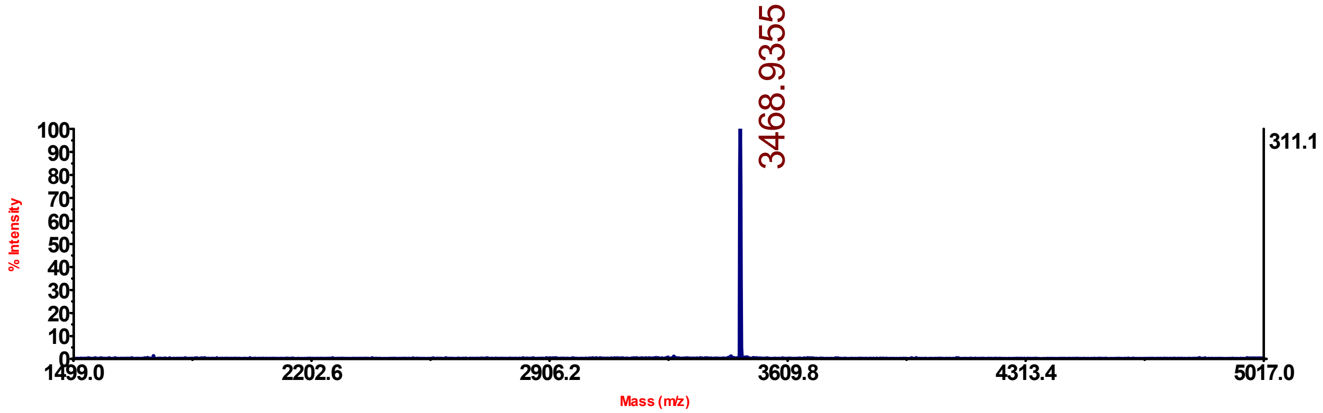

Compound **16**: MS

(a)

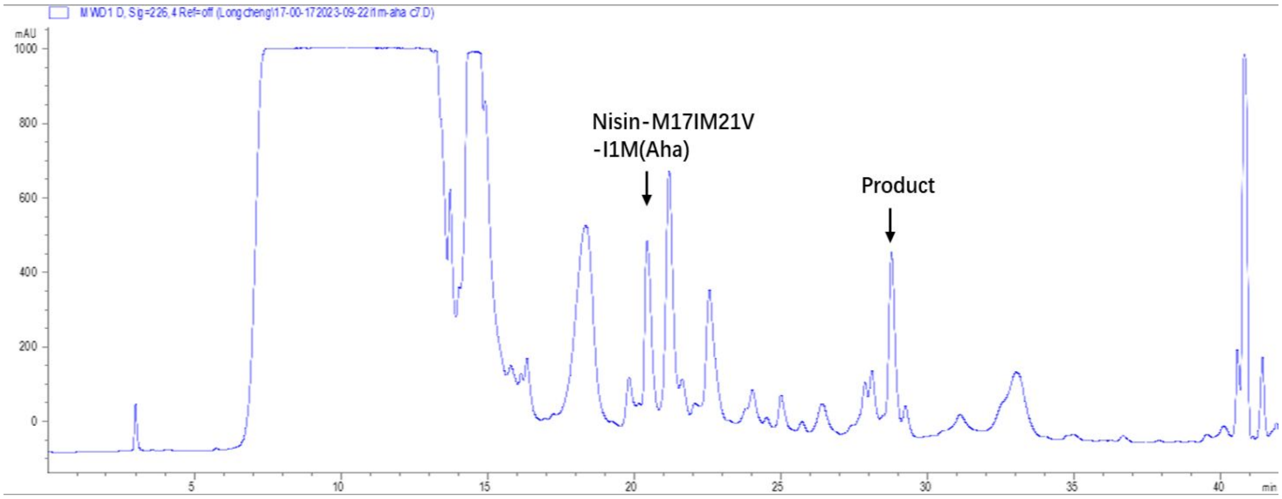

Compound **46**: Analytical HPLC

(b)

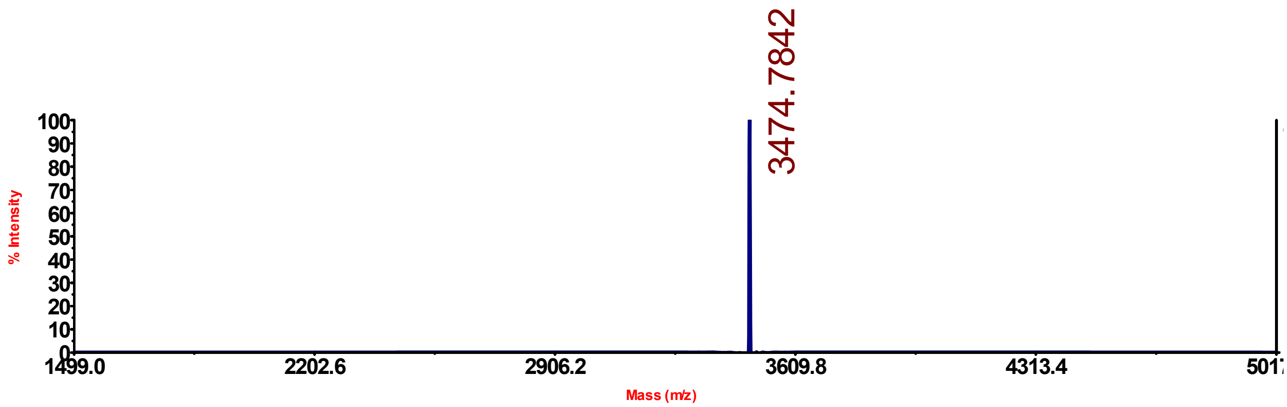

Compound **46**: MS

(a)

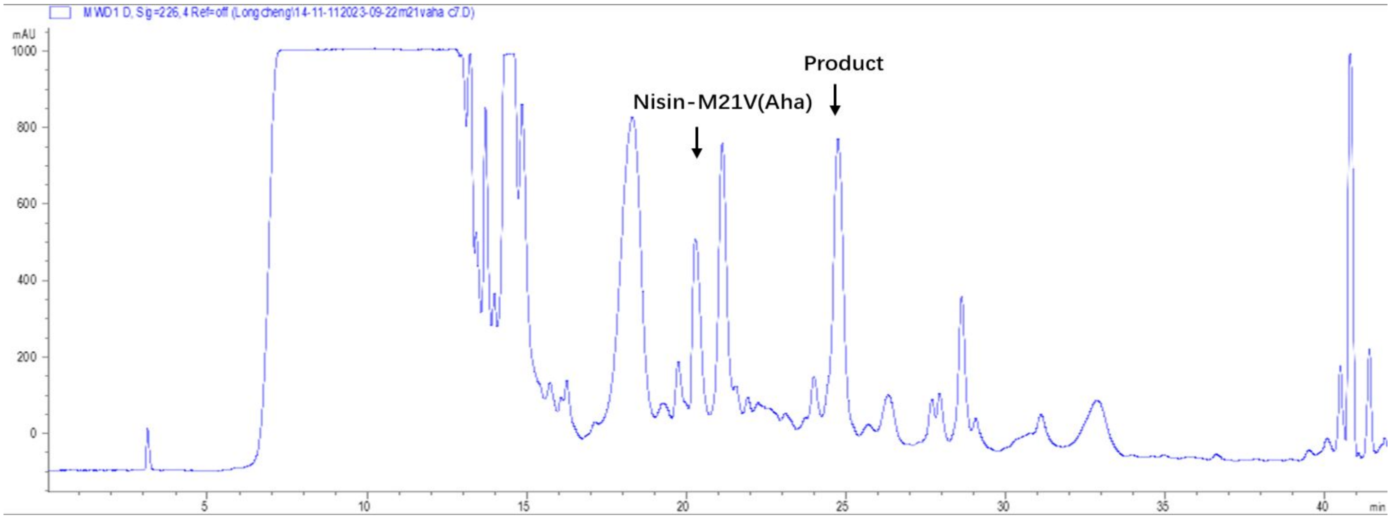

Compound **47**: Analytical HPLC

(b)

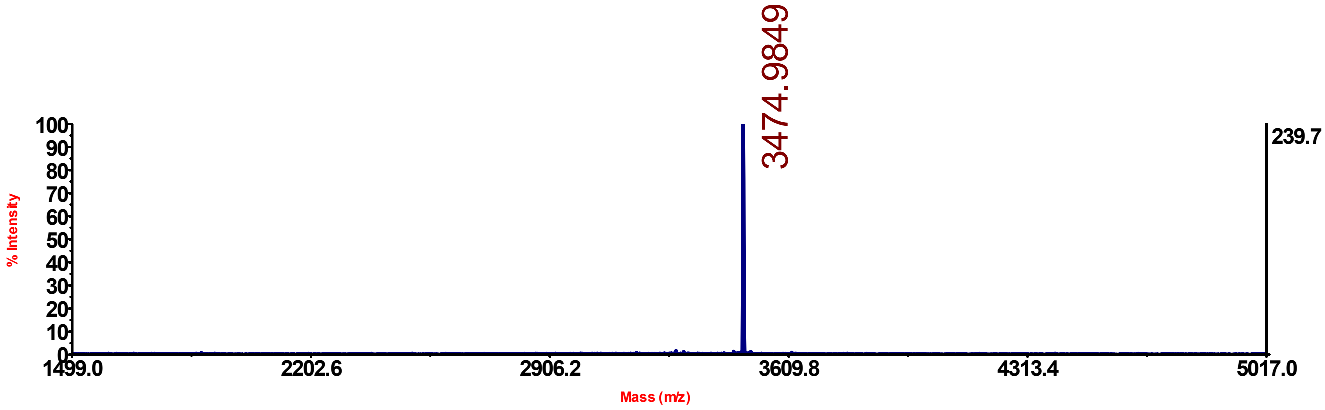

Compound **47**: MS

Figure S1. (a) Analytical high-performance liquid chromatography (HPLC) profiles of the click chemistry reaction yielding compounds **14, 16, 46, and 47.** The positions of the unmodified nisin variants and the products are indicated by an arrow. (b) MALDI-TOF mass spectra (MS) of purified nisin variants.

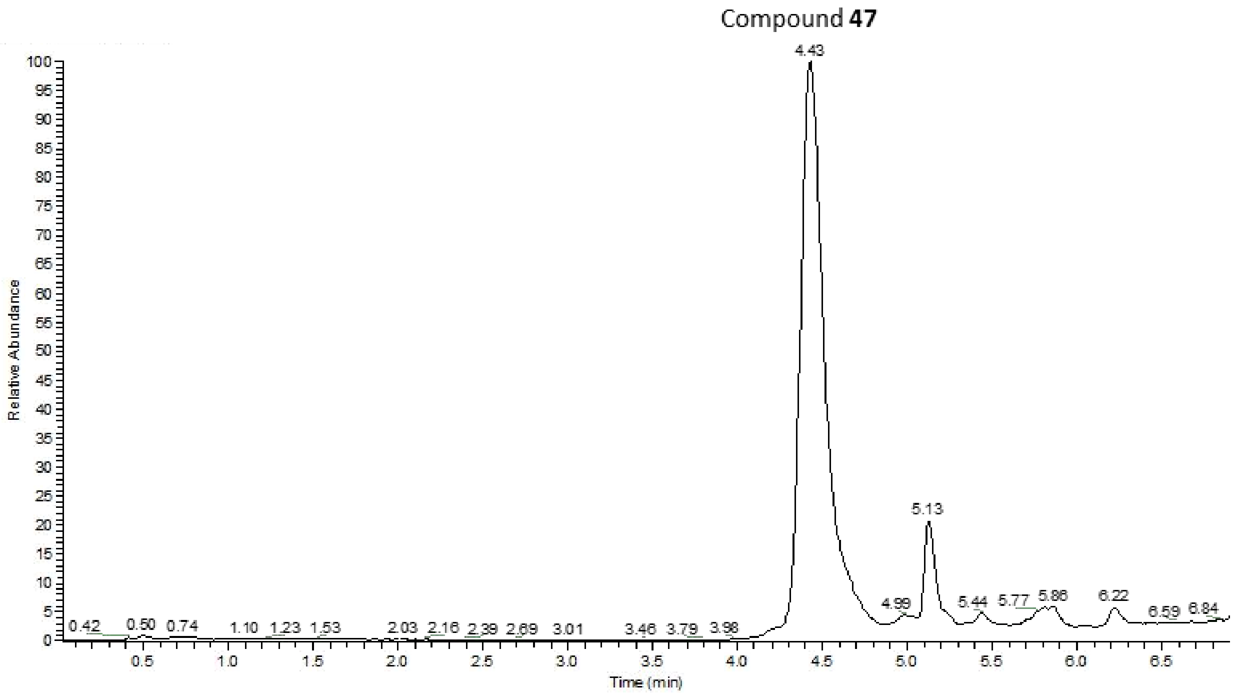

Figure S2. HPLC profile of purified compound **47**. The analysis was performed to assess the purity of compound **47**, using a Shimadzu LC20 XR-series HPLC system and an Agilent Persuit XRs C_8 column (50 × 2 mm) at 216 nm. The elution protocol utilized Eluent A (0.1% formic acid in ultra-pure water) and Eluent B (0.1% formic acid in acetonitrile), with a flow rate of 0.300 ml/min. The concentration of Pump B was 2% at 1.00 min, 45% at 5.00 min, 95% at 7.00 min, 95% at 8.00 min, and 0.5% at 8.10 min, and the analysis was stopped at 10.0 min.

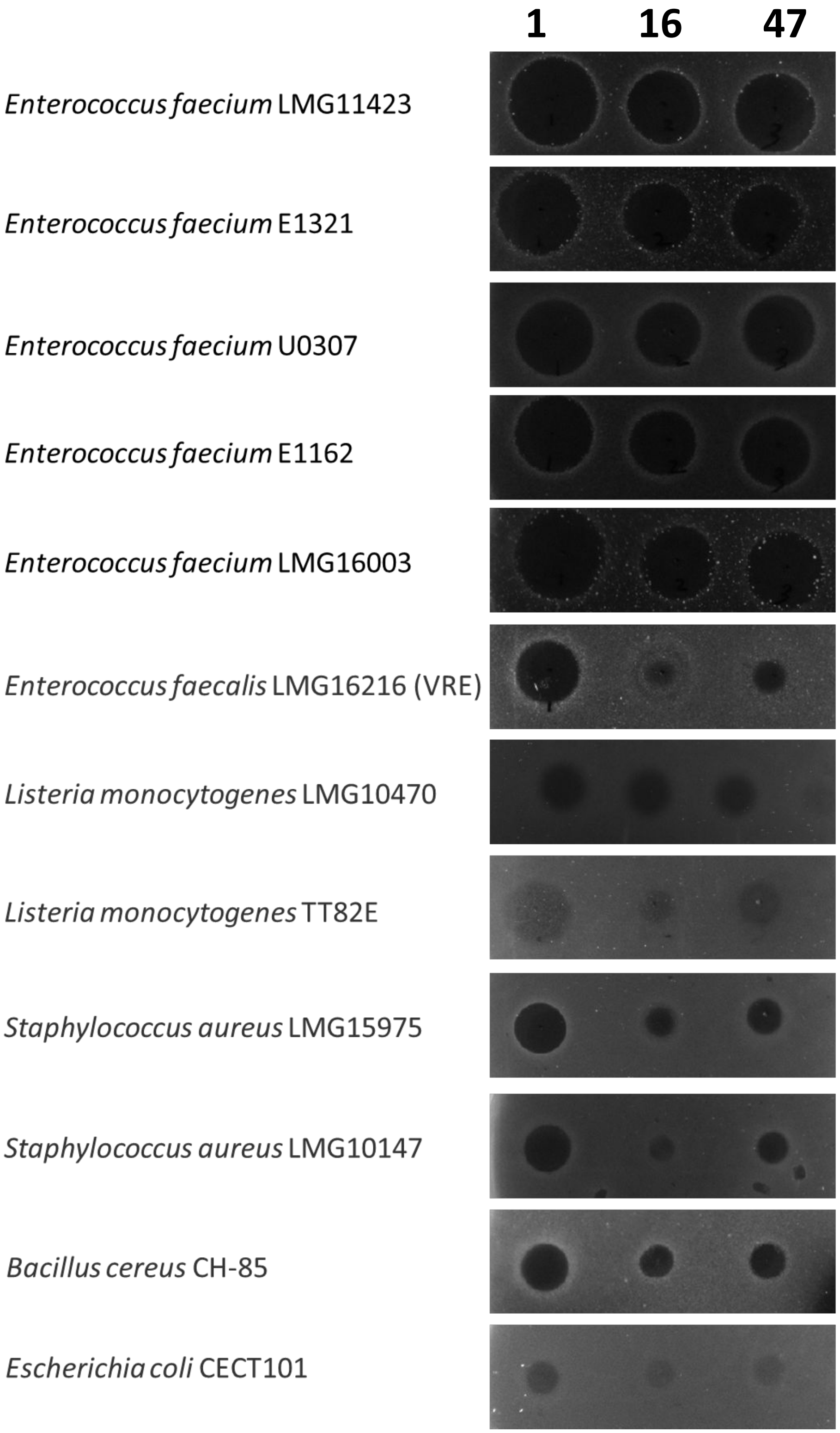

Figure S3. Spot-on-lawn assay to test the peptides antibacterial spectrum. A representative image from three independent experiments is displayed.

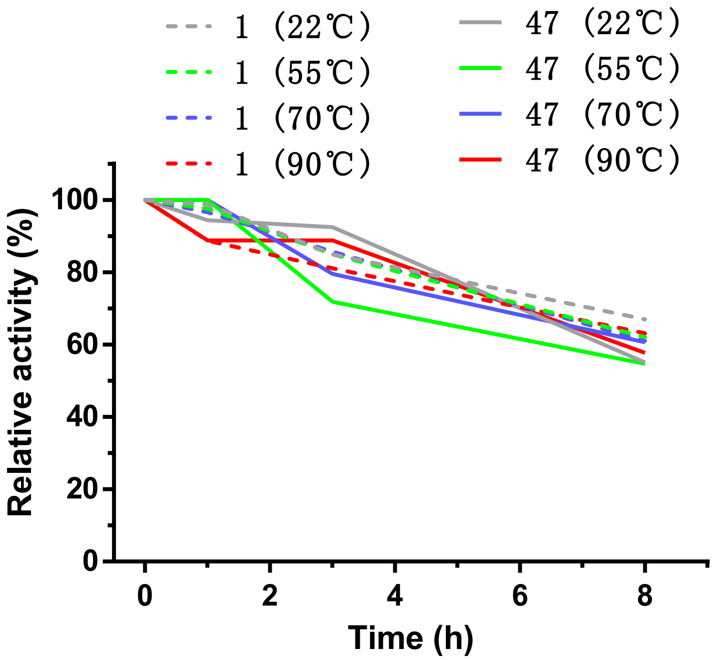

Figure S4. Investigation of the thermal stability profile of nisin (compound **1**) and compound **47**. Thermal stability was evaluated across a temperature range from 22 to 90 °C within an 8-hour incubation period. Relative antimicrobial activity of the peptides following exposure to different temperatures. The relative activity was determined by calculating the area at different time points divided by the area at the start point (labeled 0 h), multiplied by 100%. Three independent experiments were conducted.
